# Enhancement of the HIF-1α/15-LO/15-HETE Axis Promotes Hypoxia-Induced Endothelial Proliferation in Preeclamptic Pregnancy

**DOI:** 10.1371/journal.pone.0096510

**Published:** 2014-05-05

**Authors:** Dandan Yuan, Yajuan Ran, Qian Liu, Yanhua Zhang, Huiying Li, Peiling Li, Daling Zhu

**Affiliations:** 1 Department of Obstetrics and Gynecology, the Second Affiliated Hospital of Harbin Medical University, Harbin, China; 2 Department of Biopharmaceutical Sciences, College of Pharmacy, Harbin Medical University (Daqing), Daqing, China; 3 Department of Obstetrics and Gynecology, Hongqi Hospital of Mudanjiang Medical College, Mudanjiang, China; Medical Faculty, Otto-von-Guericke University Magdeburg, Medical Faculty, Germany

## Abstract

Preeclampsia (PE) is an extremely serious condition in pregnant women and the leading cause of maternal and fetal morbidity and mortality. Despite active research, the etiological factors of this disorder remain elusive. The increased release of 15-hydroxyeicosatetraenoic acid (15-HETE) in the placenta of preeclamptic patients has been studied, but its exact role in PE pathogenesis remains unknown. Mounting evidence shows that PE is associated with placental hypoxia, impaired placental angiogenesis, and endothelial dysfunction. In this study, we confirmed the upregulated expression of hypoxia-inducible factor 1α (HIF-1α) and 15-lipoxygenase-1/2 (15-LO-1/2) in patients with PE. Production of the arachidonic acid metabolite, 15-HETE, also increased in the preeclamptic placenta, which suggests enhanced activation of the HIF-1α–15-LO–15-HETE axis. Furthermore, this study is the first to show that the umbilical cord of preeclamptic women contains significantly higher serum concentrations of 15-HETE than that of healthy pregnant women. The results also show that expression of 15-LO-1/2 is upregulated in both human umbilical vein endothelial cells (HUVECs) collected from preeclamptic women and in those cultured under hypoxic conditions. Exogenous 15-HETE promotes the migration of HUVECs and in vitro tube formation and promotes cell cycle progression from the G0/G1 phase to the G2/M + S phase, whereas the 15-LO inhibitor, NDGA, suppresses these effects. The HIF-1α/15-LO/15-HETE pathway is therefore significantly associated within the pathology of PE.

## Introduction

Preeclampsia (PE) is a pregnancy-specific syndrome that seriously threatens the health and safety of mothers and children, affecting 3% to 5% of all pregnancies [1]. PE is associated with serious pathologic alterations, such as acute atherosis and diffuse vascular obstruction, including fibrin deposition, intimal thickening, necrosis, atherosclerosis, and endothelial damage [2]–[4]. Reduced placental perfusion and ischemia/hypoxia caused by impaired trophoblast invasion may induce the placenta to release various vasomotor regulatory factors, causing various angiogenic abnormalities and alterations in circulating angiogenic factors in the maternal vasculature [5], [6].

Significant morphologic changes occur in the villous architecture of the preeclamptic placenta [7]. The placentas of women with PE have been found to have a greater degree of villous capillary branching than those from healthy pregnant women, which suggests dysregulation of angiogenesis [8]. Oxygen tension is a critical factor in the regulation of angiogenesis [9]. Placental development during normal pregnancy takes place within a relatively low oxygen environment, and this physiologic hypoxia is critical for early placental development and angiogenesis [10]. In PE, the inadequate perfusion of the placenta induced by impaired trophoblast invasion leads to alterations in vascular remodeling and can cause uteroplacental hypoxia. This promotes the expression of proangiogenic factors in the placental tissue, such as hypoxia-inducible factor 1α (HIF-1α), and antiangiogenic factors including soluble fms-related tyrosine kinase 1 (sFlt-1) and soluble endoglin (sENG). Altered levels of these factors can contribute to the angiogenic imbalance associated with diseased placentas [11]–[13]. Functional interactions between anti- and proangiogenic factors have also been confirmed, suggesting mutual regulation. For example, sFlt-1 can bind to the proangiogenic growth factors vascular endothelial growth factor (VEGF) and placental growth factor (PLGF), thereby neutralizing their proangiogenic functions, resulting in insufficient angiogenesis for obtaining an adequate supply of blood and nutrients [14], [15]. Increased expression of sFlt-1 in the hypoxic placenta is also regulated by another proangiogenic factor, HIF-1α [16].

By altering the expression of angiogenic factors and their receptors, hypoxia promotes endothelial cell proliferation, migration, and formation of capillary-like tube structures [17]. In the preeclamptic placenta, increased exposure to VEGF, decreased expression of PLGF, and higher levels of the endogenous VEGF receptor inhibitor sFlt-1 and other mediators have been verified experimentally [18]. A previous study also shown that 15-hydroxyeicosatetraenoic acid (15-HETE), the arachidonic acid (AA) metabolite of 15-lipoxygenase (15-LO), is increased in the placentas of preeclamptic women [19]. During hypoxia, HIF-1α can increase the expression and activity of 15-LO and 15-HETE, eventually leading to the proliferation and migration of endothelial cells, tube-like structure formation, and inflammation [20]. Increased activity of the HIF-1α/15-LO/15-HETE functional axis can induce the constriction of human umbilical artery rings [21]. However, no studies have been performed to assess whether activating this axis affects the activity of endothelial cells that are dysregulated in PE. This study aimed to measure the placental expression of HIF-1α and 15-LO-1/2 and the placental and blood concentrations of 15-HETE in healthy and preeclamptic pregnant women. We also investigated the effects of endogenous and exogenous 15-HETE on the proliferation and migration of HUVECs. These efforts should contribute to a better understanding of the exact role of the HIF-1α/15-LO/15-HETE axis in the pathology of PE.

## Results

### Patient characteristics

Twenty participants were enrolled in this study: 10 patients with PE and 10 healthy pregnant women. The two groups of patients did not differ significantly in age, as shown in Table 1. However, the babies of the patients with PE had significantly lower birth weights, lower gestational ages on delivery, and higher systolic and diastolic blood pressures. All preeclamptic patients developed proteinuria.

**Table 1:** Basic characteristics of PE cases and normotensive controls.

Characteristics	Normal Pregnancy	PE
Number of subjects	10	10
Maternal age (y)	29.3±4.5	28.5±6.0
Gestational age (wk)	37.7±2.1	33.8±5.5*
Body Mass Index	27.7±3.0	29.0±3.7
Birth weight (kg)	3.3±0.5	2.7±1.0**
S/D ratio	2.1±0.3	3.4±1.0***
Systolic BP (mm Hg)	118.3±10.9	163.6±15.5***
Diastolic BP (mm Hg)	76.1±9.3	103.9±16.1***
Proteinuria (g/24h)	0.15±0.05	3.78±1.51***

Basic characteristics of preeclampsia cases and normotensive controls. The data are expressed as mean ± SD. PE: Preeclampsia.

*, P<0.05;

**, P<0.01;

***, P<0.001, PE vs. normal pregnancy. S/D ratio: Ratio of systolic and diastolic blood flow in the umbilical artery.

### Morphometric placental analysis and measurement of 15-LO and HIF-1α levels in placentas from healthy pregnant women and preeclamptic patients

**Figure 1:**
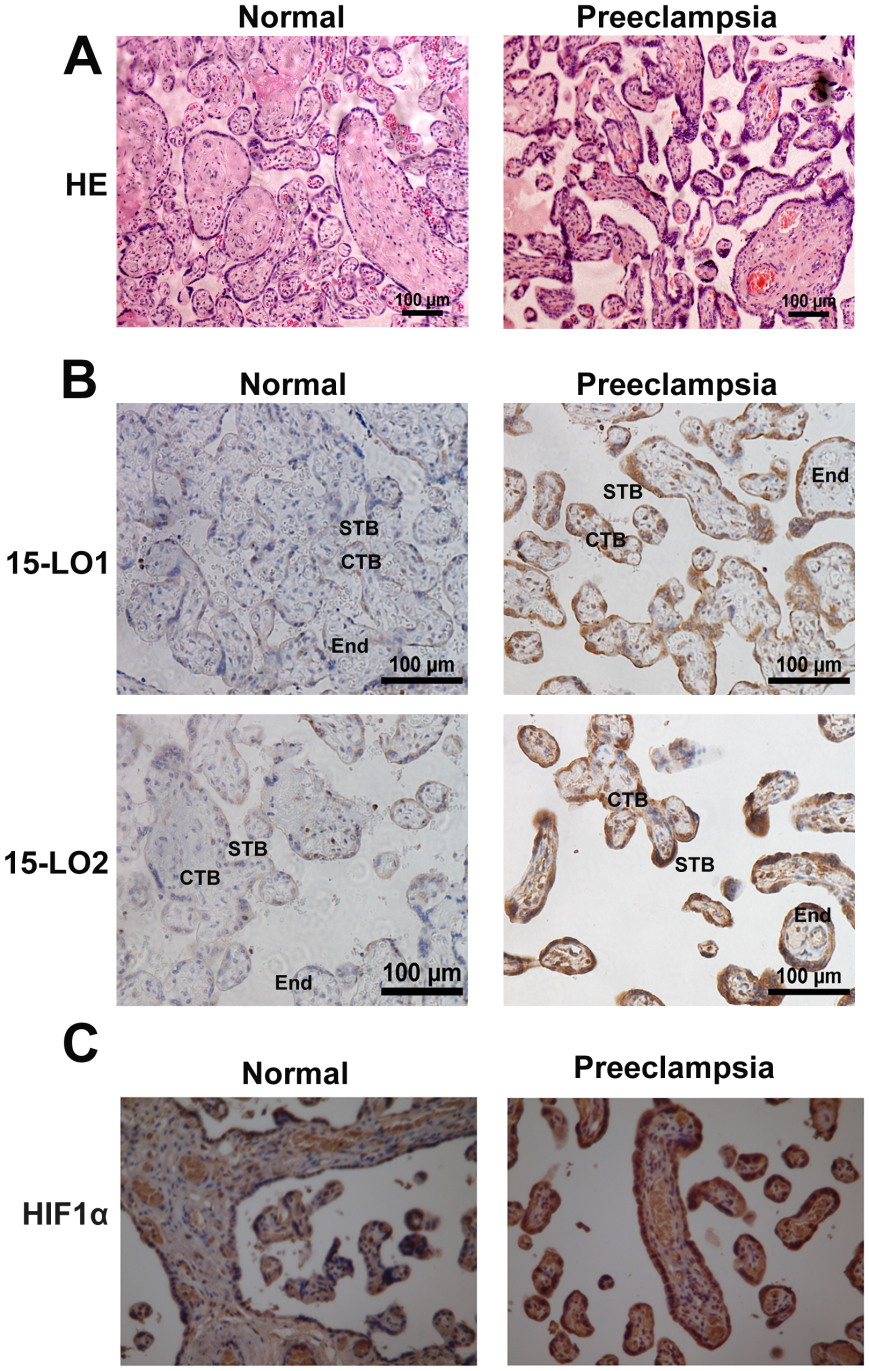
Morphologic changes in the placentas of normal and preeclamptic patients. **A.** Hematoxylin–eosin (HE) staining. CTB: cytotrophoblast, which forms the inner layer of the trophoblast; STB: syncytrophoblast, which forms the surface of the villi; End: endothelial cell, a flat cell in the villi. Localization of 15-LO-1/2 (**B**) and HIF-1α (**C**). Increased expression of 15-LO-1/2 and HIF-1α gave rise to thicker syncytiotrophoblast membranes, more syncytial knots, and thicker vessels in preeclamptic placentas than in normal placentas (n = 10).

**Figure 2:**
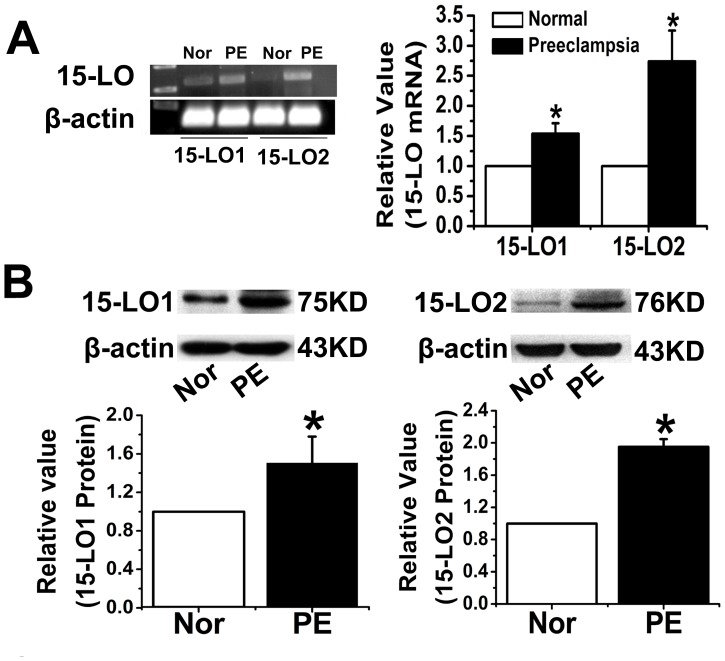
Comparisons of the gene and protein expression of 15-LO-1/2 in the placentas of normal and preeclamptic patients (n = 10). A. Result of the RT-PCR assay. PE: preeclampsia; Normal: normal pregnancies; *, P<0.05, PE compared with Normal. B. Result of the western blot analysis. *, P<0.05, PE compared with Normal.

The placenta was examined after H&E staining to determine whether there were morphologic changes potentially correlated with PE (Figure 1A). Significant changes, such as thicker syncytiotrophoblast membranes, more syncytial knots, and thicker vessel intima, were observed in the preeclamptic placentas. We also used immunocytochemical analysis to investigate the expression and localization of 15-LO-1/2 and HIF-1α in placentas from normoxic and preeclamptic patients (Figure 1B and C). The results revealed that expression of the 15-LO isozyme was significantly upregulated in preeclamptic patients, as shown by the intense staining of cytotrophoblast, syncytiotrophoblast, and endothelial cells. Expression of 15-LO-2 was also greatly upregulated (Figure 1B). A similar increase in the expression of HIF-1α was observed in preeclamptic placentas, compared to that seen in normal tissue (Figure 1C). RT-PCR and western blot analyses revealed that 15-LO-1/2 mRNA and protein expression levels increased in the placentas of preeclamptic patients relative to those of normotensive women (Figure 2).

### Placental and serum 15-HETE concentrations in normotensive and preeclamptic patients

**Figure 3:**
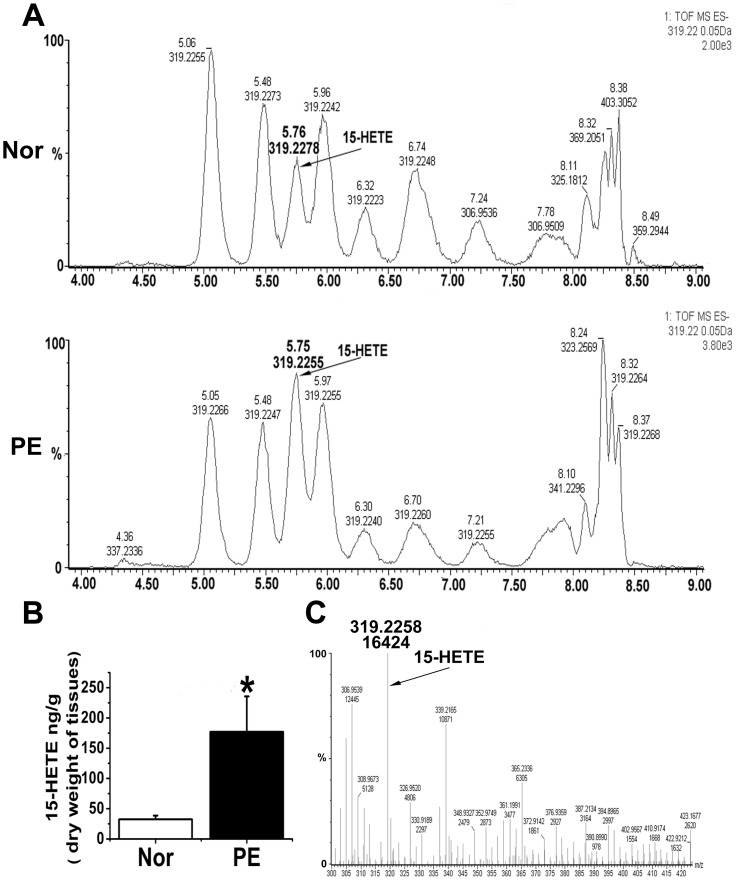
Identification and measurement of endogenous 15-HETE levels using HPLC-MS. **A.** Typical HPLC-MS chromatograms. **B**. 15-HETE content in preeclamptic placenta and normal tissue (n = 10). PE: preeclampsia; Normal: normal pregnancies; *, P = 0.03326, PE compared with Normal. **C**. Identification of 15-HETE using trap mass spectrometry. The arrow points to the 15-HETE peak in the chromatograms.

**Figure 4:**
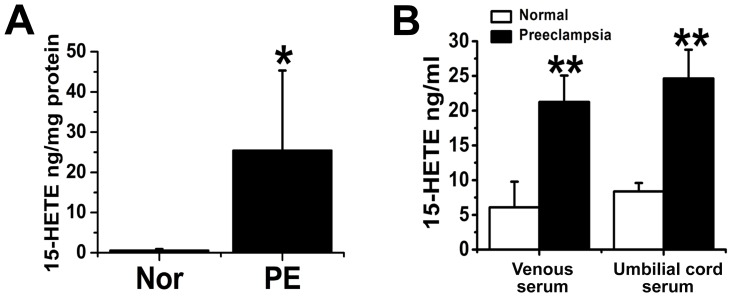
Comparisons of the levels of endogenous 15-HETE in the placentas of normal (A) and preeclamptic (B) women using a 15-HETE EIA kit (n = 10). PE: preeclampsia; Normal: normal pregnancies; *, P = 0.01066; **, P<0.01, PE compared with Normal.

Endogenous 15-HETE production in the placenta was measured using RP-HPLC and ELISA. We found that endogenous placental 15-HETE levels markedly increased in preeclamptic women (Figures 3A and 3B). We determined 15-HETE levels using trap mass spectrometry (Figure 3C). We sought to confirm these findings by ELISA. Similar results were obtained: placentas of the preeclamptic women exhibited significantly higher 15-HETE levels than those of the controls (Figure 4A). We also tested endogenous serum 15-HETE levels in venous and umbilical cord blood by ELISA. Preeclamptic venous blood contained significantly higher concentrations of 15-HETE and was majorly upregulated in umbilical cord blood (Figure 4B), suggesting that the placenta was releasing endogenous 15-HETE into the blood. The HIF-1α/15-LO/15-HETE pathway may therefore be involved in the pathogenesis of PE.

### Effect of exposure to hypoxic conditions for different durations on 15-LO-1/2 expression in HUVECs from preeclamptic and normotensive women

**Figure 5:**
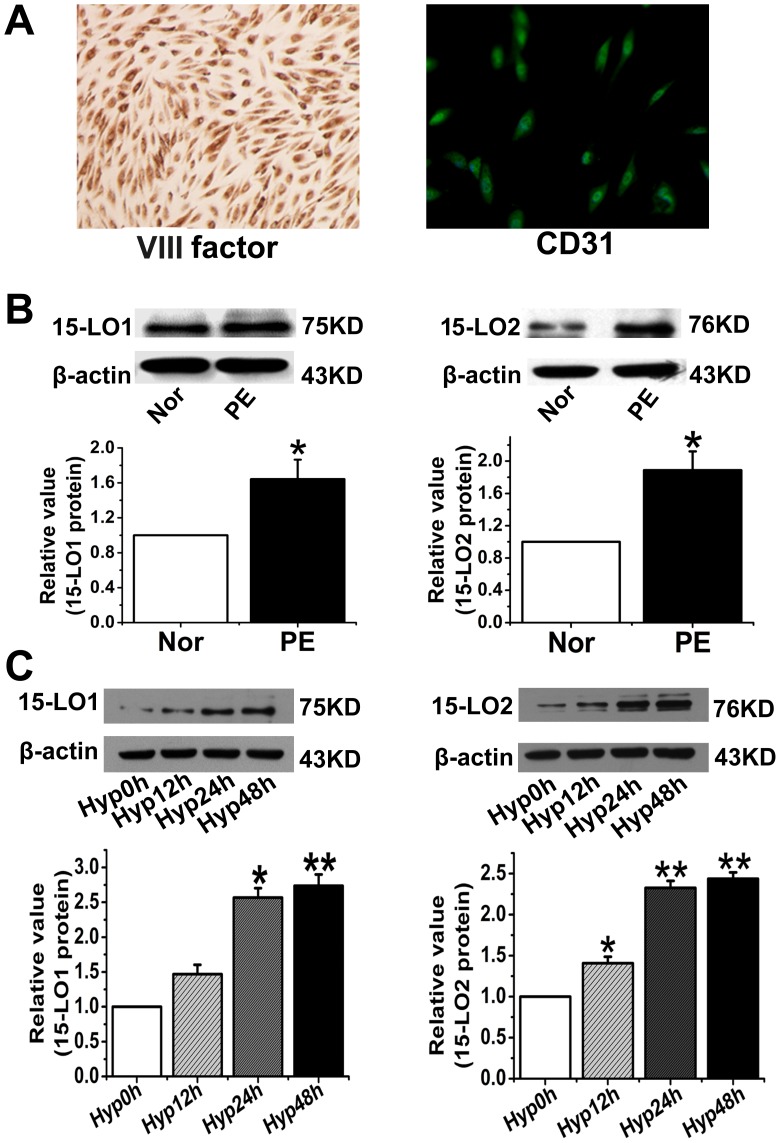
The expression of 15-LO in HUVECs. **A.** Characterization of HUVECs by VIII factor and CD31 binding. The expression of both VIII factor–related antigen and CD31 suggests the endothelial identity of the investigated cells. **B**. The expression of 15-LO in HUVECs from preeclamptic and normal placenta. n = 3; PE: preeclampsia; Normal: normal pregnancies; *, P<0.05, PE vs. Normal. **C**. 15-LO expression after exposure of HUVACs to hypoxic conditions for different durations. n = 3; Hyp: Hypoxia; **, P<0.01, compared with Hyp0h.

Endothelial cell dysfunction is important in the pathogenesis of PE [22]. HUVECs from healthy pregnant and preeclamptic patients were therefore cultured and characterized by immunocytochemical staining with VIII factor-related antigen and CD31 immunofluorescence staining, and their 15-LO levels were determined. The expression of both VIII factor-related antigen and CD31 suggested that the cells were endothelial cells (Figure 5A). We then tested 15-LO expression in primary HUVECs and found that both 15-LO-1 and 15-LO-2 were upregulated in endothelial cells from preeclamptic patients (Figure 5B). We next performed a western blot analysis to measure 15-LO protein expression in HUVECs after different periods of hypoxic exposure (0, 12, 24, and 48 h). We found that 15-LO expression increased after 12 h of hypoxia, and remained high for the rest of the study period (Figure 5C). No significant time-dependent expression alteration was found for the two proteins under normoxic conditions (Figure S1).

### Effect of 15-HETE on HUVEC viability, proliferation, migration, and tube formation

**Figure 6:**
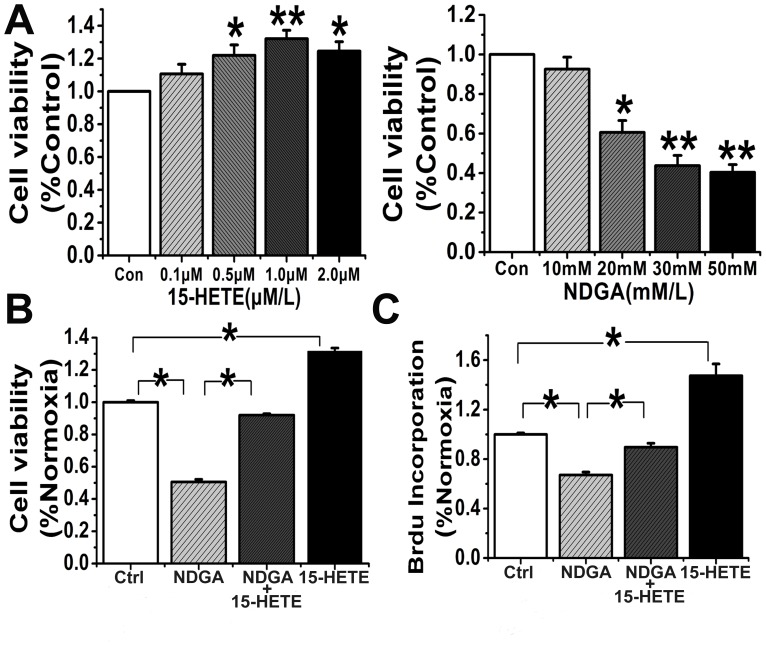
Effects of 15-HETE and NDGA on HUVEC viability and proliferation (n = 6). **A.** Effects of 15-HETE and NDGA at different concentrations ranges (0.1–2.0 µM for 15-HETE and 10–50 nM for NDGA) on the viability of HUVECs. *, P<0.05; **, P<0.01, compared with Ctrl. Effects of 15-HETE on cell viability **(B)** and proliferation **(C)** of NDGA-pretreated HUVECs were also investigated. Ctrl: untreated control; *, P<0.05, compared with Ctrl or NDGA.

**Figure 7:**
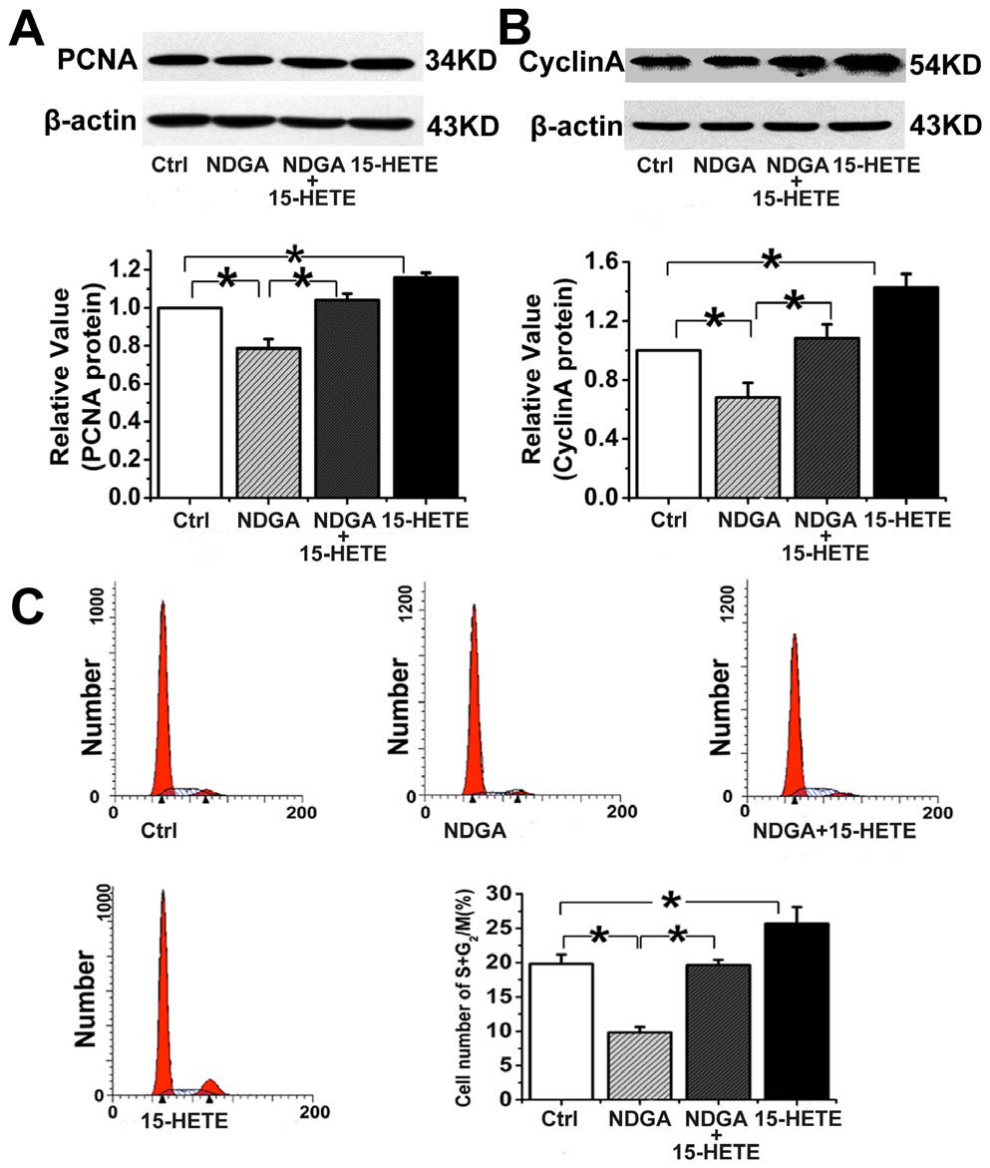
15-HETE promotion of HUVEC proliferation and cell cycle progression. Expression of PCNA (**A**) and cyclin A (**B**) proteins was determined by western blot analysis (n = 3). **C.** The percentage of cells in the G2/M+S phase detected by flow cytometry. n  = 3; *, P<0.05; Ctrl: untreated control.

Endothelial dysfunction is the cause of clinical abnormalities in PE [23]. To determine whether 15-HETE regulates endothelial cell function, we studied its effect on cell viability using a cell viability assay. First, we determined the baseline exogenous concentrations of 15-HETE and NDGA, an endogenous 15-LO inhibitor. HUVECs were treated with different doses of 15-HETE and NDGA after 24 h of growth arrest. We found that 1 µM 15-HETE significantly improved cell viability, whereas viability was significantly reduced by NDGA at 30 µM (Figure 6A). Furthermore, the NDGA-induced decrease in cell viability was reversed by exogenous addition of 15-HETE (Figure 6B). A 5-bromodeoxyuridine incorporation assay was performed to identify cells in the population that were actively synthesizing DNA. The results show that NDGA markedly inhibited cell proliferation, but exogenous 15-HETE significantly enhanced 5-bromodeoxyuridine incorporation (Figure 6C). We also detected expression of PCNA and cyclin A in HUVECs and found that NDGA significantly inhibited their expression (Figure 7A and B). In contrast, exogenous 15-HETE enhanced cell proliferation and reversed the inhibitory effects of NDGA (Figure 7A and B).

The cell cycle distribution in HUVECs was analyzed by flow cytometry to determine whether 15-HETE affects cell cycle progression. The results showed that endogenous 15-HETE increased the percentage of cells in the G2/M+S phase, whereas NDGA suppressed cell cycle progression, inducing arrest in the G0/G1 phase. The increased accumulation of cells in the G2/M+S phase in the presence of exogenous 15-HETE (Figure 7C) suggests that the 15-LO/15-HETE pathway is involved in HUVEC proliferation.

**Figure 8:**
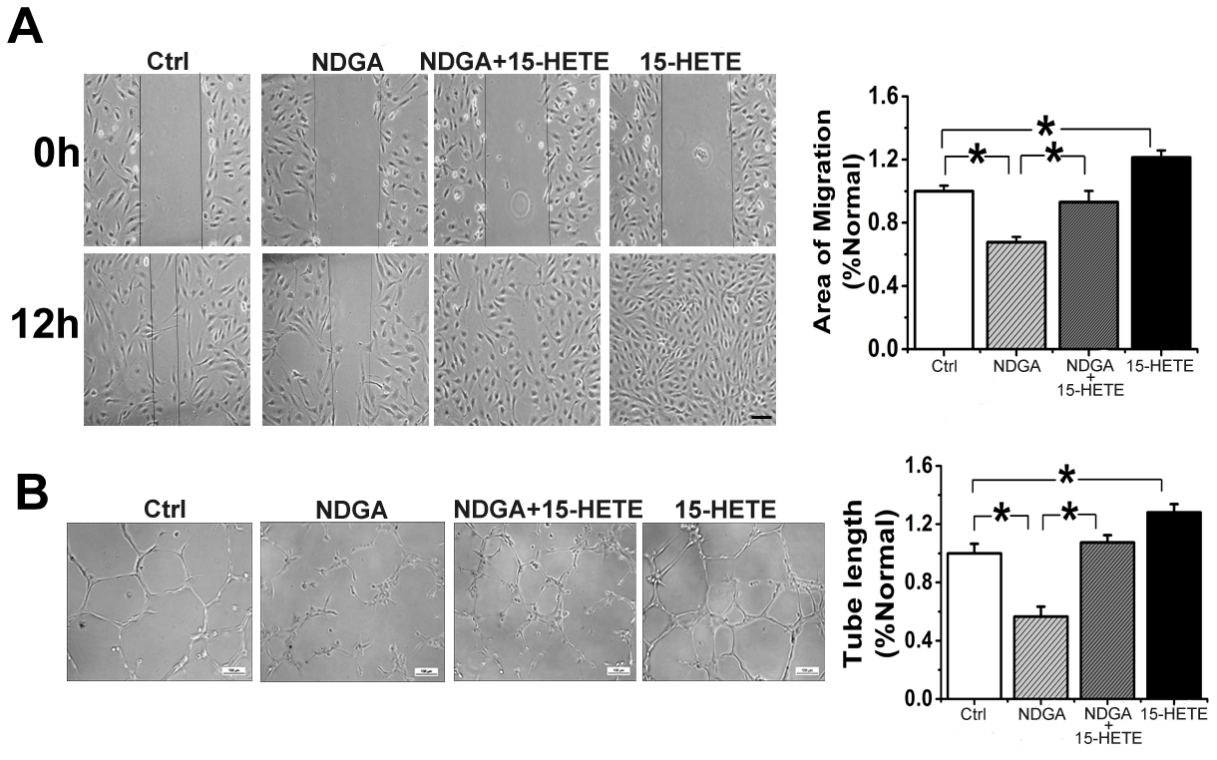
15-HETE-induced HUVEC migration (A) and tube formation (B). n = 6; Ctrl, untreated control; *, P<0.05, compared with Ctrl or NDGA.

To further examine the angiogenic effects of 15-HETE, we studied its effect on cell migration using a scratch wound assay and on tube formation in Growth Factor Reduced Matrigel. Our results show that exogenous 15-HETE stimulates HUVEC migration (Figure 8A) and tube-like structure formation (Figure 8B) as compared with untreated controls. In contrast, NDGA blocked the effect of endogenous 15-HETE on HUVEC tube formation and migration. However, this inhibitory effect was partly reversed by the addition of exogenous 15-HETE (Figure 8A and B).

## Discussion

Clinical studies suggest that PE is an independent, female-specific risk factor for early onset hypertension later in life [24]. Although early intervention may be beneficial for patients with PE, novel insights into the mechanisms underlying PE will undoubtedly deepen our understanding of its pathogenesis and provide more rational treatment strategies [25]. Numerous studies have shown that impaired angiogenesis contributes to the pathogenesis of PE, eventually leading to metabolic abnormalities in the placenta [8], [26]. Given that hypoxia-related factors such as HIF-1α are crucial in the dysregulation of angiogenesis [9], this study aimed to analyze the functional role of changes in the HIF-1α/15-LO/15-HETE axis in PE.

Consistent with previous studies [27], we detected significantly higher HIF1α expression in placental tissues from patients with PE than in those from healthy pregnant women. HIF1α overexpression was also detected in the peripheral blood of patients with PE [28]. It has been experimentally shown that antisense RNA inhibition of HIF-1α led to downregulation of transforming growth factor beta 3 (TGF-β3), restoring trophoblast differentiation and invasive capabilities, suggesting an involvement of HIF-1α in the pathogenesis of PE [29]. Taken together, we hypothesize that the HIF system may be significantly overactive in PE, which would fit with the hypoxic features of its pathology [11]. There is evidence that 15-LO can be induced by HIF-1α in hypoxic tissues [30]. This fact is in line with our finding of enhanced 15-LO mRNA and protein expression in the preeclamptic placenta. The changes in 15-LO expression, similar to those in HIF-1α, were also confirmed. Together, they suggest that HIF-1α/15-LO signaling is overactivated in PE. This finding was then confirmed in an in vitro PE model by incubating HUVECs under hypoxic conditions. Long-term hypoxia clearly increased the HUVEC 15-LO-1/2 protein levels.

A serious consequence of HIF-1α/15-LO signal overactivation is the sharply increased production of 15-HETE in preeclamptic placental tissues. Extremely high 15-HETE concentrations were also detected in the venous and umbilical cord sera of patients with PE. Excess 15-HETE, far beyond physiological levels, may cause a series of functional changes, including cell proliferation and migration, and even constriction of the umbilical arteries [15], [16]. We also investigated the effects of exogenous 15-HETE on HUVECs under normoxic conditions. These experiments showed that exogenous 15-HETE administration to HUVECs clearly increased the expression of two proteins, PCNA and cyclin A, that are highly sensitive markers for cell proliferation, suggesting the important role of 15-HETE in promoting cell proliferation and migration. The specific 15-LO inhibitor, NDGA, reversed these effects of exogenous 15-HETE. Our results are therefore consistent with previous work indicating the important role of overactivation of the HIF-1α/15-LO/15-HETE axis in the pathophysiology of PE. However, there is a potential contradiction implicit in our findings of pathologic activation of the functional axis and impaired placental angiogenesis in PE. Taken with the results of previous studies [31], [32], it suggests that an imbalance between anti- and proangiogenic factors may be one of the main mechanisms underlying the pathogenesis of PE. Our results suggest that, in PE, upregulation of theupregulated HIF-1α/15-LO/15-HETE axis with its angiogenic potential exists in nature to counter-balance the simultaneous lack of angiogenesis caused by the neutralization ofneutralized proangiogenic factors such as sFlt-1 and sENG. Multiple alterations in altered signaling pathways may occur to offset conflicting effects at the same time as feedback mechanisms are in operation [33]. Relevant to this, two recent clinical studies the reported that increased serum levels of sFlt-1 and sENG and decreased serum VEGF and PlGF all failed to accurately predict the risk of developing PE [34], [35], indicating that additional markers are needed to be identified to develop a panel of predictive markers for clinical utility. Further studies are definitely needed to determine the integrated effects of different angiogenesis-related factors dysregulated in PE.

In conclusion, PE is a pregnancy-specific syndrome that causes hypertension. The current lack of effective treatments prompts demand for a better understanding of the mechanisms underlying PE. Our study confirms the overactivation of the HIF-1α/15-LO/15-HETE axis in PE and reports the key effects of this overactivation on the proliferation and migration of endothelial cells, contributing to a more comprehensive understanding of PE pathogenesis. However, several limitations exist in our study, such as significant gestational age difference between PE patients and healthy pregnancies and higher O_2_ concentration used in normoxic experiment. We will investigate whether these affect our results in our future study.

## Materials and Methods

### Reagents

Nordihydroguaiaretic acid (NDGA), 15-HETE, 15-LO-1, and 15-LO-2 polyclonal antibodies were purchased from Cayman Chemical Company (Ann Arbor, MI, USA). Growth Factor Reduced Matrigel and the CycleTEST PLUS DNA Reagent Kit were obtained from BD Biosciences (Bedford, MA, USA). The bromodeoxyuridine (BrdU) proliferation assay kit was purchased from the Millipore Corporation (Billerica, MA, USA). Antibodies against β-actin, CD31, cyclin A, and proliferating cell nuclear antigen (PCNA) were purchased from Santa Cruz Biotechnology Inc. (Santa Cruz, CA, USA). All other reagents were from common commercial sources.

### Sample collection

All participants provided their written informed consent. Umbilical cord, placental, and venous blood samples were obtained from healthy and preeclamptic pregnant women during caesarian deliveries at the Second Affiliated Hospital of Harbin Medical University, using procedures approved by the Harbin Medical University Ethical Committee for the Use of Human Samples. Healthy pregnant participants were defined as those with blood pressures <140/90 mmHg and with no clinically significant complications. Patients with preeclamptic hypertension were defined as those with blood pressure ≥140/90 mmHg after 20 weeks into pregnancy and significant proteinuria. Immunoturbidimetric assay was used to measure the total amount of proteinuria within 24 h. Patients with chronic hypertension were excluded from the study. Pregnant woman experiencing uterine contractions before caesarian delivery were also not included in our study in order to avoid the uncertain effect of placental hypoxia caused by contractions.

### Isolation and culture of HUVECs

Umbilical cords (15 cm to 20 cm in length) were excised from the placenta immediately after delivery and placed into cold sterile phosphate-buffered saline (PBS) with double resistance. HUVECs were acquired from the umbilical cord by the method of Jaffe et al [36], and cultured as described by Lampugnani et al [37]. Normoxic or hypoxic (93% N_2,_ 5% CO_2_, 2% O_2_) conditions were applied for cell incubation using a Tri-Gas incubator (HF100; Heal Force, China).

### Histology

Placental tissues obtained from caesarian deliveries were sectioned into blocks and immersed in 4% paraformaldehyde for overnight fixation. The fixed tissues were then dehydrated, cleared, and embedded in paraffin wax. They were then cut into 5-μm-thick sections and stained with hematoxylin and eosin (H&E), before being viewed under a Nikon Eclipse 600 microscope and photographed with a digital camera.

### Morphometric analysis

The immunohistochemical methods used have been described by Reilly et al [38]. After normal serum was used to bind nonspecific sites, the sections were incubated with anti-15-LO-1 (1∶50), anti-15-LO-2 (1∶200), or anti-HIF-1α (1∶400) antibody. Parallel controls were incubated with PBS. After overnight incubation, the sections were washed three times with PBS and then probed with secondary antibodies from the corresponding species at a 1∶200 dilution. Then, the sections were stained with 3,3′-diaminobenzidine (DAB) and counterstained with hematoxylin. Brown and yellow staining indicated the sections were positive for HIF-1α, 15-LO-1, or 15-LO-1. A ranking-based standard was used for quantification of protein expression. Briefly, brown and yellow granules in a cell nucleus or cytoplasm represented positive cells. Five fields were randomly selected on each slice at high magnification (×400), and 1000 cells were counted (200 cells/field). Negative (-) was defined as the presence of less than 1% positive cells; weakly positive (+), as 1–10% positive; moderately positive (++), as 10–50% positive; and strongly positive (+++), as over 50% positive.

### MTT assay

HUVECs were cultured in 96-well microtitration plates (about 1 × 10^4^ cells per well). The cells were subjected to growth arrest for 24 h, and then treated every 24 h with NDGA (30 µM), 15-HETE (1 µM), 15-HETE plus NDGA, or ethanol (vehicle) in M199 with 5% FBS. After 48 h incubation at 37 °C, the cells were incubated for 4 h in medium containing 0.5% 3-[4,5-dimethylthiazol-2-yl]-2,5-diphenyl-terazolium bromide (MTT). The amount of blue formazan dye formed from MTT is proportional to the number of surviving cells. The reaction was continued until DMSO was added to the medium, followed by incubation for 10 min at 37 °C. The absorbance was read at 540 nm using a spectrophotometer.

### Western blot analysis

HUVECs were cultured as for the MTT assay. Briefly, after 24 h at 37 °C, the cells were gently washed three times with cold PBS, treated in 300 µL of lysis buffer (50 mM Tris, pH 7.4, 150 mM NaCl, 1% Triton X-100, 1 mM EDTA, and 2 mM PMSF), and then cooled for 30 min on ice. The lysates were sonicated on ice and then centrifuged at 12,000 rpm for 10 min at 4 °C, and the insoluble fraction was discarded. The supernatants were collected and stored at −20 °C until used for western blot analyses. Placenta tissues collected from the umbilical cord were lysed in RIPA buffer (150 mM sodium chloride, 1.0% NP-40 or Triton X-100, 0.5% sodium deoxycholate, 0.1% SDS, 50 mM Tris, pH 8.0) with manual extraction of total placental protein. Protein concentrations were determined by the Bradford assay using bovine serum albumin (BSA) as the standard. Protein samples (20 µg) were fractionated by SDS-PAGE (7.5% to 10% polyacrylamide gels). Primary antibodies against 15-LO-1, 15-LO-2, PCNA, and cyclin A were used, with β-actin as the internal control.

### Reverse transcription-polymerase chain reaction (RT-PCR)

Frozen tissue samples were sealed with liquid nitrogen and then disrupted and homogenized with a Polytron homogenizer. Total RNA was extracted using Trizol reagent and its concentration determined by ultraviolet spectrophotometry (absorbance at 260 nm/280 nm). Isolated RNAs were reverse transcribed using a Superscript first-strand cDNA synthesis kit. The cDNA samples were then amplified in a DNA thermal cycler. The primer sequences are given below:

15-LO-1 forward: 5′-TGGAGCCTTCCTAACCTACAGC-3′

15-LO-1 reverse: 5′-ATGGTGACAAAGTGGCAAACC-3′

15-LO-2 forward: 5′-CGCTGTCACTACCTCCCAAAGA-3′

15-LO-2 reverse: 5′-CAACCAGTCCCACTTGTCATCAG-3′

β-actin forward: 5′-CATGTACGTTGCTATCCAGGC-3′

β-actin reverse: 5′-CTCCTTAATGTCACGCACGAT-3′

The PCR products were run on a 1% agarose gel and stained by ethidium bromide. The images were recorded and band intensities measured using a gel imaging analysis system, using β-actin mRNA levels as the internal standard to normalize 15-LO-1/2 expression. The ratios of the optical density (OD) of the target 15-lipoxygenase isozymes to β-actin were determined.

### Bromodeoxyuridine incorporation

The HUVECs were cultured in 96-well plates (about 1 × 10^4^ cells for each well) and then subjected to growth arrest for 24 h in M199 medium without growth factor and FBS, before treatment with different agents in 5% FBS M199 medium, as described in the MTT assay. BrdU proliferation assay kits were used to measure BrdU incorporation according to the manufacturer's protocol. Absorbance was measured at 450 and 540 nm.

### Tube formation assay

Each 96-well culture plate was coated with Growth Factor Reduced Matrigel (BD Biosciences, USA) in a volume of 35 µL/well and allowed to polymerize for 30 min at 37 °C. HUVECs were trypsinized, resuspended, and 200 µL of cell suspension (about 8000 cells) added into each well, as previously described [39]. The vehicle and other reagents were added to the wells and the cells incubated at 37 °C for another 24 h. Tube formation was observed under an inverted microscope (Nikon, Japan). Tube length was measured using the Image Pro Plus 6.0 software.

### Scratch-wound assay

HUVECs were cultured in 6-well plates. After confluency was achieved, the cells were wounded with pipette tips and the dead cells washed off with PBS. Subsequently, the cells were treated with the appropriate vehicle or drugs. The wounded areas were photographed after 0, 12, and 24 h incubation using a Zeiss confocal microscope (LSM 510 Meta Axiovert 200 M). The Zeiss software was programmed to capture an image of each well at the same wound location every 30 minutes for 15–19 hours, and the rate of migration assessed by measurements of wound width.

### Immunocytochemical assay

Isolated HUVECs were cultured on polylysine-coated coverslips in 24-well plates. The cells were washed with cold PBS after incubation for 24 h at 37 °C, fixed with 4% paraformaldehyde for 10 min, treated with 3% hydrogen peroxide for 15 min at room temperature to block intrinsic peroxidase activity, washed three times with PBS, and then blocked in 1% BSA for 30 min. The cells were incubated overnight at 4 °C with VIII factor-related antigen. Subsequently, the cells were washed three times with PBS and incubated with secondary IgG for 1 h at 37 °C. The cells were stained with DAB and counterstained with hematoxylin. Brown and yellow staining indicated that the cells were positive for VIII factor-related antigen.

### Immunofluorescence assay

HUVECs were placed onto coverslips that were covered with polylysine in 24-well culture plates. After incubation for 24 h at 37 °C, the cells were washed with cold PBS, fixed with 4% paraformaldehyde for 10 min, and blocked in 1% BSA for 30 min. The cells were incubated at 4 °C overnight with anti-CD31 (1∶100) antibody. After the cells were washed three times with PBS, they were incubated at 37 °C for 2 h in the dark with FITC-conjugated secondary antibodies (1∶100) diluted in PBS, and with 4′,6-diamidino-2-phenylindole (DAPI). The slides were then examined under a microscope (Nikon, Japan), and the images recorded by digital photomicrography (Olympus, Japan). Green staining indicated the cells were positive for CD31.

### Cell cycle and DNA analyses

A CycleTEST PLUS DNA Reagent kit was used to determine if cell cycle progression in HUVECs was influenced by endogenous and exogenous 15-HETE. After treatment with vehicle and reagents, the cells were harvested with trypsin and fixed in 70% ethanol. After removing the ethanol and incubating the cells with 200 µL of PBS, they were stained with propidium iodide according to the manufacturer's protocol. DNA fluorescence was measured, and flow cytometry was performed using a BD FACSCalibur Flow Cytometer (Becton Dickinson, Bedford, MA, USA). For each sample, 2 × 10^4^ events were plotted in a histogram. The distribution of cells in the different phases of the cell cycle was calculated from each histogram.

### Measurement of 15-HETE levels by reverse-phase high-pressure liquid chromatography (RP-HPLC) and mass spectrometry

Frozen tissue samples were thawed on ice, and the wet tissues were accurately weighed (about 0.06 g). Then, 100 µL EtOH and liquid N_2_ were added. The tissues were pulverized into fine pulp and transferred to a fresh tube using 4 × 200 µL EtOH washes. The internal standard, prostaglandin B1 (PGB1, 100 µL 100 pmol/mL EtOH solution), HCOOH (2 µL, 88% v/v), and BHT (antioxidant; 0.5 µL, 0.5% w/v EtOH solution) were added to the sample, which was given high-speed shocks for a few seconds. After mixing, the sample was left on ice for 1 h to extract the arachidonic acid metabolites and then centrifuged at 150,000 rpm for 20 min at 4 °C. The supernatant was transferred to another fresh tube and diluted with 3 mL of distilled water. This was then applied to a Strata-X polymeric SPE column (200 mg/6 mL) that had been preconditioned with 5 mL of 100% EtOH followed by 5 mL of 25% EtOH. The column was then washed with 12 mL of distilled water, 5 mL of 25% EtOH, and 6 mL of distilled water and allowed to run dry. Thereafter, the eicosanoids were eluted with EtOAc containing 5 mL of 0.0002% BHT. After discarding the water from the organic solution, the tube containing the remaining EtOAc layer was placed in an ice bath and dried by flushing with N_2_. After about 1 h, the dried sample was reconstituted in 300 µL of 100% EtOH as the test solution. Approximately 100 µL of test solution was added to the second internal standard, 15-HETE (3 µL, 800 pg/mL EtOH solution) as the reference solution. Finally, test and reference solutions were transferred to sample vials for UPLC-TOF analysis.

UPLC analysis was performed on a Waters Acquity UPLC system using a Waters Acquity BEH C18 column (2.1 mm × 50 mm column, 1.7-μm particle size) maintained at 30 °C. The sample injection volume was 5 µL, and the sample injector was maintained at 4 °C throughout the analysis. The mobile phase consisted of 0.05% aqueous formic acid (B) and methanol/acetonitrile (1∶4, v/v) (A) at a flow rate of 0.25 mL/min. The mobile phase gradient was run from 30% A to 100% A in 7 min, returned to 30% A in 1.5 min, and maintained at 30% A for 1.5 min for re-equilibration. A TOF mass spectrometer equipped with an electrospray ionization (ESI) interface (Waters, LCT premier XE) was used for the MS experiments. ESI was performed in the negative ionization mode with the capillary voltage set to 2300 V, sample cone set to 100 V, and the desolvation and source temperatures set to 200 °C and 110 °C, respectively. The desolvation gas flow rate was set to 700 L/h, and the cone gas flow to 30 L/h. The peak at 319.2252 was the [15HETE-H]^−^ in the negative ionization mode.

To ascertain endogenous serum and placental 15-HETE levels, a 15(S)-HETE EIA Kit (catalog no. 534721, Cayman, USA) was used. Placental tissues were homogenized by sonication and ground with a mortar and pestle. Then, 0.1 M Tris–HCl (pH 7.4) with 1 × 10^−3^ M EDTA and 1 × 10^−5^ M indomethacin were added to the homogenized tissue on ice. Serum was obtained from venous and umbilical cord blood. Endogenous 15-HETE was then measured with the kit. Protein concentrations were determined with the Bradford assay. The results were analyzed using Cayman Chemical Company Enzyme Immunoassay (EIA) Tools.

### Statistical analysis

The data were expressed as mean ± SEM from at least three independent experiments. Statistical analysis was performed with the Student's *t*-test or one-way ANOVA, followed by a Dunnett's test, where appropriate. Differences were only considered significant at *p*<0.05.

## Supporting Information

Figure S1 15-LO expression of HUVACs under normoxic conditions for different durations (n = 3). Nor: normoxia. No significant expression alteration was found.
(TIF)
