# Supplementary figures and images for: Enhancement of the HIF-1α/15-LO/15-HETE Axis Promotes Hypoxia-Induced Endothelial Proliferation in Preeclamptic Pregnancy
Source: PLoS One. 2014 May 5;9(5):e96510. doi: 10.1371/journal.pone.0096510 (PMC4010521; doi:10.1371/journal.pone.0096510)

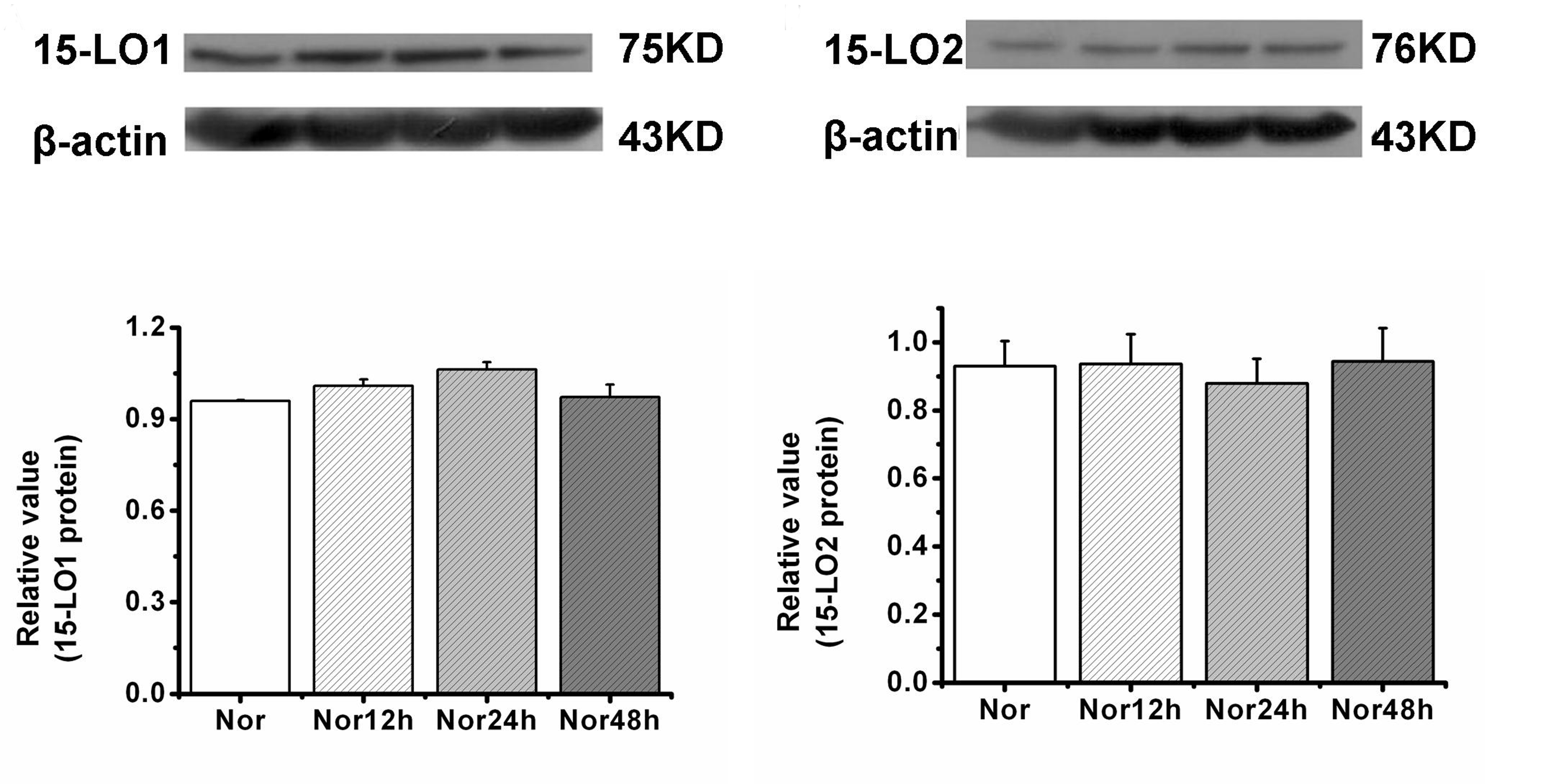

Supplement: Figure S1 — 15-LO expression of HUVACs under normoxic conditions for different durations (n = 3). Nor: normoxia. No significant expression alteration was found. (TIF) [file pone.0096510.s001.tif]
